# High rates of death and loss to follow-up by 12 months of rifampicin resistant TB treatment in South Africa

**DOI:** 10.1371/journal.pone.0205463

**Published:** 2018-10-09

**Authors:** Kamban Hirasen, Rebecca Berhanu, Denise Evans, Sydney Rosen, Ian Sanne, Lawrence Long

**Affiliations:** 1 Health Economics and Epidemiology Research Office, Department of Internal Medicine, School of Clinical Medicine, Faculty of Health Sciences, University of the Witwatersrand, Johannesburg, South Africa; 2 Division of infectious diseases, University of North Carolina, Chapel Hill, United States of America; 3 Department of Global Health, School of Public Health, Boston University, Boston, MA, United States of America; 4 Right to care, Johannesburg, South Africa; 5 Clinical HIV Research Unit, Department of Internal Medicine, School of Clinical Medicine, Faculty of Health Sciences, University of the Witwatersrand, Johannesburg, South Africa; Indian Institute of Technology Delhi, INDIA

## Abstract

**Introduction:**

Treatment success rates of rifampicin resistant (RR)/multi-drug resistant (MDR) tuberculosis (TB) in South Africa range from 43–48%, falling short of the World Health Organization’s target of ≥75%. We present rates and assess predictors of attrition by 12 months on treatment.

**Methods:**

Prospective observational cohort analysis of adults (≥18 years) initiating RR/MDR-TB treatment from 01 March 2013 to 30 September 2016. Attrition was defined as a combination of death and loss to follow-up (LTFU; treatment interruption ≥2 months) by 12 months on treatment. Predictors of attrition were identified using Cox Proportional Hazards models to estimate crude (HR) and adjusted hazard ratios (aHR) with corresponding 95% confidence intervals.

**Results:**

By 12 months on treatment, 75/240 (31.3%) patients had either died (37/240; 15.4%) or been LTFU (38/240; 15.8%). Patients with moderate/severe anaemia (aHR: 2.10; 95% CI 1.00–4.39), and those who were smear positive at baseline (aHR: 2.04; 95% CI 1.01–4.12) were significantly more likely to die or be lost from care.

**Conclusion:**

At this outpatient DR-TB treatment site, there was a high rate of attrition halfway through the standard treatment course at 12 months of 31%. High rates of attrition by 12 months on treatment may continue during the second-half of therapy.

## Introduction

South Africa, with the highest tuberculosis (TB) incidence in the world (781/100 000[1]) as well as the largest HIV epidemic[2], faces a substantial drug-resistant TB (DR-TB) burden of 19 073 cases of laboratory-diagnosed rifampicin (RIF) resistance in 2016[1].

In a bid to improve overall TB case detection and identify rifampicin drug resistance (RR), in March 2012 South Africa implemented Xpert MTB/RIF (Cepheid, Sunnyvale CA), a rapid molecular assay used for the detection of mycobacteria tuberculosis (MTB) and RR as the first-line diagnostic test for TB[3, 4]. Following the roll-out of Xpert MTB/RIF, RR/multi-drug resistant (MDR) TB case detection more than doubled, from 10 085 in 2011 to a peak of 26 023 cases in 2013[5].

Prior to 2012 all RR/MDR-TB cases were treated at specialist inpatient facilities for the duration of the intensive phase of treatment (generally 6–8 months)[6]. However, a shortage of inpatient beds, the high cost of inpatient treatment, and patient reluctance to be hospitalized resulted in delays in time to treatment initiation pre-treatment MDR-TB mortality and loss to follow-up (LTFU)[7–9].

In an attempt to reduce treatment initiation delays and treatment costs as well as to improve treatment access and outcomes, the South African National TB Programme (NTP), implemented a policy of “decentralized and deinstitutionalized treatment” of DR-TB in 2011[10]. Under this policy, eligible patients could start treatment in an outpatient TB clinic and make regular visits to primary health clinics (PHC).

The parallel implementation of Xpert MTB/RIF and decentralization of treatment have resulted in a substantial increase in the numbers of patients accessing treatment[11] and significant reductions in time to treatment initiation[6, 12]. Disappointingly, despite these improvements and significant programmatic achievements, treatment outcomes for RR/MDR-TB remain poor, with high rates of on-treatment mortality and LTFU limiting treatment success[13]. The current treatment success rate of RR/MDR-TB in South Africa is 43–48%, falling far short of the World Health Organization’s (WHO) target of ≥75%[14].

We have previously described the high rates of early mortality (9%) and LTFU (14%) by 6 months at a single decentralized DR-TB treatment site in Johannesburg, South Africa[6]. We now report on the interim, 12-month outcomes of RR/MDR-TB treatment and aim to assess predictors of attrition (death and LTFU) by 12 months.

## Methods

### Design

We conducted a prospective observational cohort study of patients with RR-TB (regardless of resistance to other TB drugs) at an outpatient decentralized hospital-based TB clinic in Johannesburg, South Africa.

### Study population

**Fig 1:**
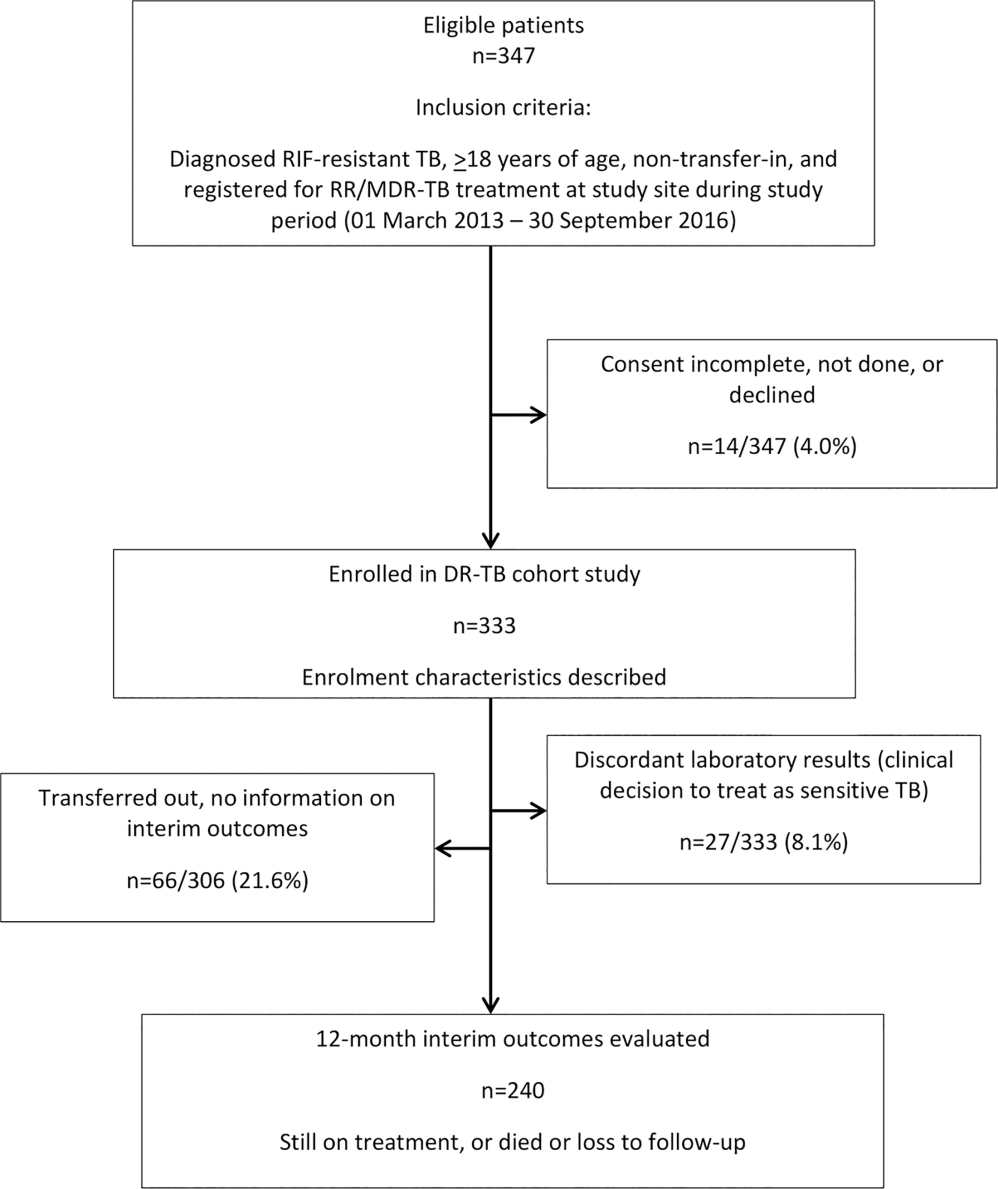
Description of cohort.

We included adult patients (≥18 years) who initiated RR/MDR-TB treatment between 01 March 2013–30 September 2016. Complicated cases or patients with extensively drug-resistant TB (pre-XDR/XDR-TB) (i.e. additional resistance to aminoglycosides and/or fluoroquinolones respectively) were not eligible for the study and were referred to Sizwe Tropical Diseases Hospital for inpatient treatment. Patients who transferred to another facility during the first 12 months of treatment (no outcome available), were excluded from the outcomes analysis. Likewise, patients who had transferred-in to the study site after receiving ≥1 month of DR-TB treatment from another site as well as those with discordant laboratory results (clinical decision to treat as sensitive after initial positive resistance testing) were also excluded (Fig 1).

### Site

The TB Focal Point (TBFP) is an outpatient TB clinic located within the tertiary-level, public academic Helen Joseph Hospital, in Johannesburg, South Africa. Under the South African NTP, the clinic became a decentralized DR-TB treatment facility providing comprehensive integrated HIV and TB care in 2012[10, 15].

### Data and variables

We used an in-house electronic patient medical record using data that is prospectively collected, regularly checked for accuracy and completeness, and managed on-site by clinic and study staff.

Treatment outcomes were measured by 12 months post RR/MDR-TB treatment initiation. Due to the relatively small number of patients having experienced death or LTFU by this time, these outcomes were combined into our primary outcome, namely attrition by 12 months. Baseline demographic and clinical variables were evaluated as risk factors for attrition (Table 1). We defined baseline as the closest to RR/MDR-TB treatment initiation (from +/- 2 months of RR/MDR-TB treatment initiation), apart from viral load (among those on ART >6 months) and CD4 cell count tests, in which case baseline was defined as the closest result between 12 months before and 14 days after RR/MDR-TB treatment initiation in keeping with national antiretroviral therapy (ART) guidelines[16]. Time to treatment initiation was defined among patients referred to treatment from outpatient facilities as the time between the initial diagnostic test and RR/MDR-TB treatment start.

**Table 1:** Baseline demographic and clinical characteristics of DR-TB patients at enrolment (n = 333) and of patients included in 12 month interim outcomes analysis (n = 240).

		Enrolled* (n = 333)	Included in analysis (n = 240)
Variable	Description	n (%)	n (%)
**Sex**	Male	169/333 (50.8%)	127/240 (52.9%)
	Female	164/333 (49.3%)	113/240 (47.1%)
**Age at Initiation (years)**	Median (IQR)	36.0 (29.0–42.0)	36.0 (29.0–42.0)
	18–29	96/333 (28.8%)	62/240 (25.8%)
	30–49	202/333 (60.7%)	151/240 (62.9%)
	≥50	35/333 (10.5%)	27/240 (11.3%)
**Employment**	Employed	153/331 (46.2%)	109/238 (45.8%)
	Unemployed	178/331 (53.8%)	129/238 (54.2%)
**Year of treatment initiation**	2013	94/333 (28.2%)	74/240 (30.8%)
	2014	109/333 (32.7%)	72/240 (30.0%)
	2015	71/333 (21.3%)	51/240 (21.3%)
	2016	59/333 (17.7%)	43/240 (17.9%)
**HIV Status**	Negative	52/333 (15.6%)	32/240 (13.3%)
	Positive	278/333 (84.2%)	208/240 (86.7%)
	Unknown	3/333 (0.9%)	0/240 (0.0%)
**CD4 Cell Count (cells/mm**^**3**^**)**	Median (IQR)	88.5 (27.0–234.5)	91.0 (27.0–218.0)
	≤50	92/260 (35.4%)	66/197 (33.5%)
	51–100	47/260 (18.1%)	37/197 (18.8%)
	>100	121/260 (46.5%)	94/197 (47.7%)
**ART Status**	On ART	133/269 (49.4%)	104/203 (51.2%)
	Not on ART	136/269 (50.6%)	99/203 (48.8%)
**Time on ART (months)**	Median (IQR)	12.7 (4.3–40.1)	13.8 (4.3–42.3)
	≥6	88/131 (67.2%)	71/103 (68.9%)
	<6	43/131 (32.8%)	32/103 (31.1%)
**Viral Load (copies/mL)**	Median (IQR)	399.0 (203.0–53212.0)	399.0 (146.0–53212.0)
	<1000	20/34 (58.8%)	15/27 (55.6%)
	≥1000	14/34 (41.2%)	12/27 (44.4%)
**Diabetes**	No	308/319 (96.6%)	226/234 (96.6%)
	Yes	11/319 (3.5%)	8/234 (3.4%)
**Haemoglobin, g/dL (Anaemia)**†	Median haemoglobin (IQR)	10.6 (8.7–12.3)	10.6 (8.7–12.4)
	Normal haemoglobin	48/251 (19.1%)	34/179 (19.0%)
	Mild anaemia	95/251 (37.9%)	70/179 (39.1%)
	Moderate anaemia	66/251 (26.3%)	47/179 (26.3%)
	Severe anaemia	42/251 (16.7%)	28/179 (15.6%)
**BMI**^‡^ **(kg/m**^**2**^**)**	Median (IQR)	22.0 (19.8–24.6)	21.7 (19.8–24.3)
	Low (<18.5)	42/263 (16.0%)	30/190 (15.8%)
	18.5–25	164/263 (62.4%)	125/190 (65.8%)
	25–30	39/263 (14.8%)	25/190 (13.2%)
	≥30	18/263 (6.8%)	10/190 (5.3%)
**Referring Facility**	Outpatient	213/333 (64.0%)	154/240 (64.2%)
	Inpatient	120/333 (36.0%)	86/240 (35.8%)
**Time to Treatment Initiation (days)**	Median (IQR)	9.0 (6.0–38.5)	9.0 (6.0–36.0)
	≤7	89/212 (42.0%)	63/153 (41.2%)
	>7	123/212 (58.0%)	90/153 (58.8%)
**Patient Category**	New–no prior history of TB	215/330 (65.2%)	151/238 (63.5%)
	Previously treated, 1^st^ line drugs	102/330 (30.9%)	76/238 (31.9%)
	Previously treated, 2^nd^ line drugs	13/330 (3.9%)	11/238 (4.6%)
**Resistance Pattern**	RIF-resistant by Xpert MTB/RIF, unconfirmed	103/330 (31.2%)	68/240 (28.3%)
	RIF mono-resistant (INH sensitive)	135/330 (40.9%)	109/240 (45.4%)
	MDR-TB (RIF and INH resistant)	92/330 (27.9%)	63/240 (26.3%)
**TB Type**	PTB^§^ + EPTB^ǁ^	43/333 (12.9%)	33/240 (13.8%)
	EPTB only	26/333 (7.8%)	22/240 (9.2%)
	PTB only	264/333 (79.3%)	185/240 (77.1%)
**Smear Microscopy**	Negative	235/313 (75.1%)	185/227 (81.5%)
	Positive	78/313 (24.9%)	42/227 (18.5%)

*Enrolled patients include those who were excluded from the 12 month outcome analysis (i.e. those who transferred out or were treated as drug-sensitive TB due to discordant test results)

†None/normal (Hb≥13g/dL for males; Hb≥12g/dL for females), †mild (10g/dL<Hb<13g/dL for males; 10g/dL<Hb<12g/dL for females), †moderate (8g/dL≤Hb≤10g/dL for males and females) and †severe anemia (Hb<8g/dL for males and females)

^‡^BMI: Body Mass Index

^§^PTB (Pulmonary TB)

^ǁ^EPTB (Extra-pulmonary TB)

### Statistical analysis

The demographic and clinical characteristics of patients included in the analysis were presented using medians with corresponding interquartile ranges (IQR) for continuous variables while categorical variables were presented as simple proportions. Kaplan Meier survival analysis was used to determine the timing of death, LTFU and attrition in the first 12 months of treatment. Loss to follow-up was defined as treatment interruption ≥2 months from the last scheduled clinical visit, with no subsequent visit. Considering the potential time variance of the primary outcome observed factors associated with attrition were identified using Cox proportional hazards models to produce crude (HR) and adjusted hazard ratios (aHR) with corresponding 95% confidence intervals (CI). Person-time accrued from RR/MDR-TB treatment initiation until earliest of death, LTFU, or 12 months on treatment. Variables *a priori* (sex, age, HIV and ART status) or with a p-value <0.10 in crude analysis were included in the multivariate model. Schoenfeld residuals were used to test the assumption of proportional hazards. Adjusted models were assessed using complete case analysis. All analyses were carried out using SAS version 9.4 (SAS Institute, Cary, North Carolina, USA).

### Ethical consideration

Ethical approval was granted by the Human Research Ethics Committee at the University of the Witwatersrand (protocol number: M130205). Patients provided written informed consent for study participation. The study is presented according to the STROBE checklist for observational cohorts[17].

## Results

### Description of cohort

During the study period, 347 patients initiated RR/MDR-TB treatment and met study eligibility criteria. Of these, 14 (4.0%) did not provide informed consent, had incomplete consent documented or declined to participate, leaving 333 enrolled in the study. Twenty seven patients (27/333; 8.1%) with discordant RIF-resistance results who were subsequently treated as drug sensitive TB were excluded from the outcomes analysis as were 66 patients (66/306; 21.6%) who transferred to a different treatment facility and for whom 12-month outcomes could not be assessed. There remained 240 patients in the final analytic cohort (Fig 1).

### Baseline demographic and clinical characteristics

Table 1 presents the baseline demographic and clinical characteristics of patients enrolled in the study (n = 333) and those included in the final analytic cohort (n = 240). Baseline characteristics were similar between all patients enrolled and those included in the analytic cohort (Table 1). Of those successfully enrolled, approximately half were female (164/333; 49.3%). Median age at treatment initiation was 36.0 years (IQR: 29.0–42.0), with approximately two thirds of patients between 30–49 years (202/333; 60.7%).

The majority of patients were HIV co-infected (278/333; 84.2%), with a low median CD4 cell count of 88.5 cells/mm^3^ (IQR: 27.0–234.5) and more than half (139/260; 53.5%) having a CD4 cell count ≤100 cells/mm^3^ at RR/MDR-TB treatment initiation. Close to half of HIV positive patients were already on ART when starting RR/MDR-TB treatment (133/269; 49.4%), but 40% of the patients on ART for 6 months or more had a detectable viral load (≥1000 copies/mL (14/34; 41.2%)). Median haemoglobin at start of treatment was 10.6 g/dL (IQR: 8.7–12.3), with close to 50% of patients having either moderate (66/251; 26.3%) or severe anaemia (42/251; 16.7%).

Over one third of patients in the cohort were diagnosed with RR/MDR-TB while hospitalized (120/333; 36.0%); the rest were diagnosed at an outpatient clinic facility. Median time from diagnosis to treatment initiation was 9.0 days (IQR: 6.0–38.5) with less than half of patients initiating within 1 week (89/212; 42.0%). The proportion of positive smear microscopy results was slightly higher among those enrolled than in those included in the outcomes analysis (24.9% vs. 18.5%; p>0.05).

### Outcomes 12 months after RR/MDR-TB treatment initiation

**Fig 2:**
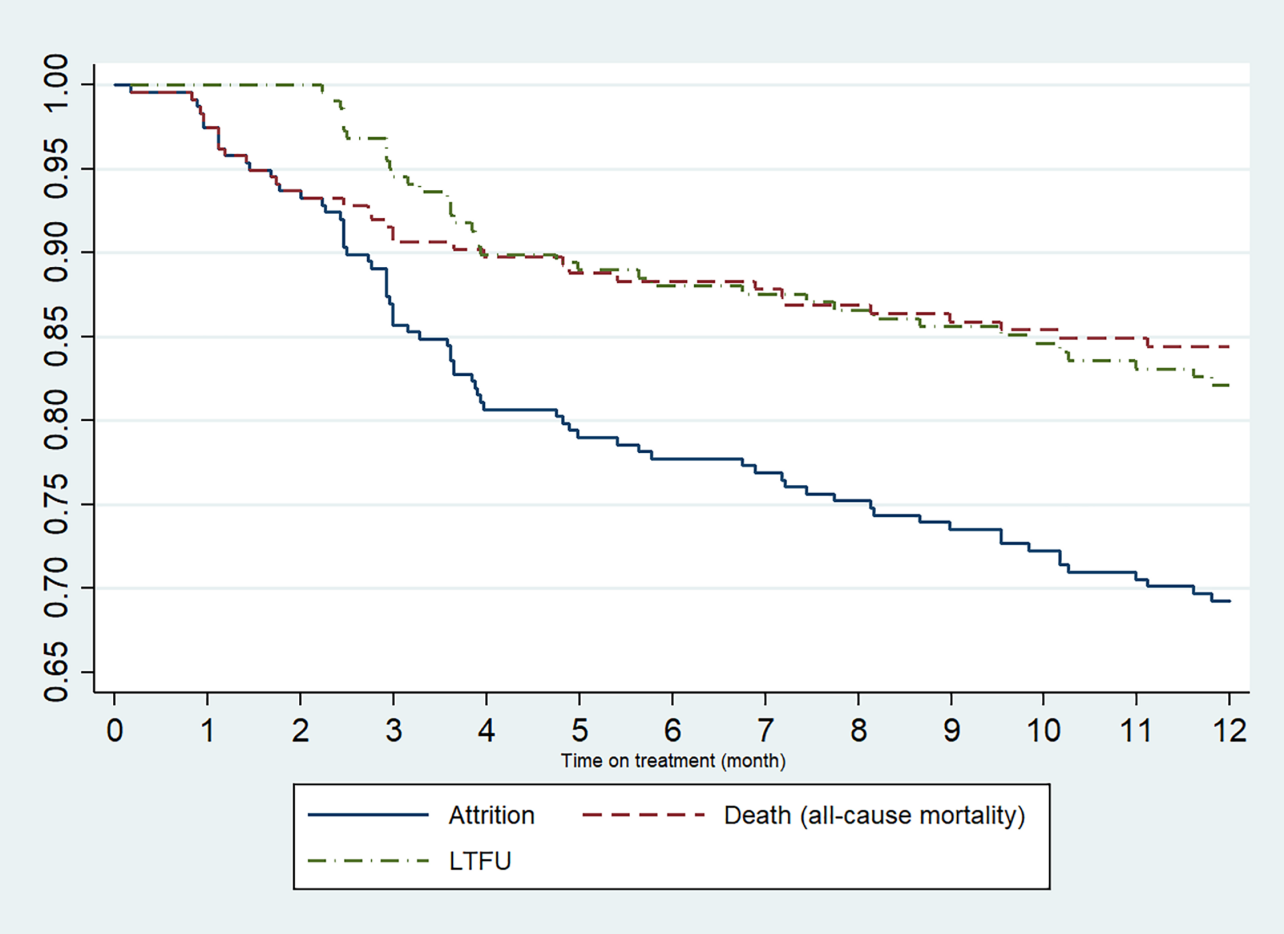
Kaplan-Meier survival estimate of treatment outcomes by 12 months post RR/MDR-TB treatment initiation.

Fig 2 presents the Kaplan-Meier estimates of death, LTFU, and total attrition by 12 months after RR/MDR-TB treatment initiation. By 12 months on treatment, a total of 75/240 (31.3%) patients had either died (37/240; 15.4%) or been LTFU (38/240; 15.8%). Median time to death was 2.59 months (IQR: 1.11–5.15) and LTFU was 3.85 months (IQR: 2.92–7.74). Rates of death and LTFU were highest during the first three months of treatment but persisted over time (Fig 2).

### Predictors of 12-month attrition

Table 2 presents the crude and adjusted hazard ratio estimates of attrition (sum of death and LTFU) at 12 months by baseline demographic and clinical characteristics. In crude analyses; patients with moderate/severe anaemia (vs. none/mild anaemia; HR: 2.61; 95% CI: 1.57–4.32), those referred to treatment from outpatient facilities who initiated treatment more than 7 days from initial diagnostic testing (outpatient clinic >7 days to treatment vs. outpatient clinic ≤7 days to treatment; HR: 2.34; 95% CI: 1.11–4.97), as well as those who were smear positive (vs. smear negative; HR: 1.90; 95% CI: 1.10–3.27) were more likely to experience attrition by 12 months. Adjusted analyses revealed moderate/severe anaemia (vs. none/mild anaemia; aHR: 2.10; 95% CI: 1.00–4.39) as well as positive smear microscopy (vs. smear negative; aHR: 2.04; 95% CI: 1.01–4.12) as significant predictors of attrition by 12 months on treatment.

**Table 2:** Unadjusted and adjusted estimates of the relationship between demographic and clinical characteristics and attrition (death + LTFU) of DR-TB patients by 12 months (n = 240).

Variable	Description	n (row %)	Crude HR (HR)	p-value	Adjusted HR (aHR)	p-value
**Sex**	Male	33/127 (26.0%)	1		1	
	Female	42/113 (37.2%)	1.57 (0.99–2.49)	0.06	1.48 (0.7.3–301)	0.28
**Age at Initiation (years)**	18–29	21/62 (33.9%)	1.30 (0.77–2.20)	0.33	1.48 (0.68–3.19)	0.32
	30–50	43/151 (28.5%)	1		1	
	≥50	11/27 (40.7%)	1.64 (0.84–3.19)	0.15	1.04 (0.30–3.61)	0.95
**Employment**	Employed	31/109 (28.4%)	1		1	
	Unemployed	43/129 (33.3%)	1.12 (0.70–1.79)	0.62	-	-
**HIV & ART Status**	Negative	7/32 (21.9%)	1		1	
	Positive on ART	34/104 (32.7%)	1.64 (0.73–3.70)	0.23	1.37 (0.40–4.64)	0.61
	Positive not on ART	32/99 (32.3%)	1.48 (0.65–3.37)	0.35	0.96 (0.28–3.27)	0.95
**Diabetes**	No	68/226 (30.1%)	1		1	
	Yes	3/8 (37.5%)	1.30 (0.41–4.12)	0.66	-	-
**Anaemia**^**†**^	None/mild	27/104 (26.0%)	1		1	
	Moderate/severe	38/75 (50.7%)	2.61 (1.57–4.32)	0.00*	2.10 (1.00–4.39)	0.05*
**BMI**^‡^ **(kg/m**^**2**^**)**	Normal/high (≥18.5)	40/160 (25.0%)	1		1	
	Low (<18.5)	12/30 (40.0%)	1.79 (0.91–3.49)	0.09	1.28 (0.55–3.00)	0.57
**Referring Facility & Time to Treatment**	Outpatient, ≤7 days	10/63 (15.9%)	1		1	
	Outpatient, >7 days	29/90 (32.2%)	2.34 (1.11–4.97)	0.03*	1.34 (0.50–3.56)	0.56
	Inpatient	36/86 (41.9%)	3.41 (1.64–7.07)	0.00*	1.58 (0.60–4.19)	0.35
**Patient Category**	New	45/151 (29.8%)	1		1	
	Previously treated	30/87 (34.5%)	1.19 (0.74–1.89)	0.47	-	-
**Resistance Pattern**	RIF-resistant by Xpert MTB/RIF, unconfirmed	25/68 (36.8%)	1		1	
	RIF mono-resistant (INH sensitive)	31/109 (28.4%)	0.76 (0.44–1.30)	0.32	-	-
	MDR-TB (RIF and INH resistant)	19/63 (30.2%)	0.81 (0.44–1.49)	0.50	-	-
**TB Type**	PTB^§^ + EPTB/EPTB^ǁ^ only	16/55 (29.1%)	1		1	
	PTB only	59/185 (31.9%)	1.09 (0.63–1.90)	0.76	-	-
**Smear Microscopy**	Negative	46/185 (24.9%)	1		1	
	Positive	19/42 (45.2%)	1.90 (1.10–3.27)	0.42*	2.04 (1.01–4.12)	0.05*

*p≤0.05

^†^None/normal (Hb≥13g/dL for males; Hb≥12g/dL for females), †mild (10g/dL<Hb<13g/dL for males; 10g/dL<Hb<12g/dL for females), †moderate (8g/dL≤Hb≤10g/dL for males and females) and †severe anemia (Hb<8g/dL for males and females)

^‡^BMI: Body Mass Index

^§^PTB: Pulmonary Tuberculosis

^ǁ^EPTB: Extra-pulmonary Tuberculosis

Schoenfeld residuals global test = 0.50 (p>0.05 –proportionality assumption satisfied)

## Discussion

This study examined RR/MDR-TB treatment outcomes at a single decentralized facility in Johannesburg, South Africa 12 months after treatment initiation, approximately halfway through the prescribed standard 18–24 month long-course regimen in use at the time of the study. Twelve-month mortality in this cohort of patients was 15%. Loss to follow up was also substantial at 16%. Overall attrition thus reached 31% by 12 months post RR/MDR-TB treatment initiation. Our 12-month results are already similar to other studies from the region, where attrition by 24 months (combined outcome of death and LTFU) ranged from 20% to 46%, with variability between regions of the country and models of care delivery[12, 18–20]. Moreover, the attrition rates seen at 12 months in this study are nearly equal to the global average of 17% death and 15% LTFU (32% attrition) by 24 months reported by the WHO in 2016[21].

We found that patients who had moderate or severe anaemia or who were smear-positive at treatment initiation to be more likely to experience death or LTFU. In adjusted analyses, moderate/severe anaemia was associated with a twofold increase in the risk of death or LTFU. This likely reflects the association between anaemia of chronic inflammation and advanced TB and HIV disease[22, 23]. Numerous studies have identified anaemia at TB diagnosis to be associated with poorer treatment outcomes[24–27]. Patients with smear positive pulmonary disease were also more likely to die or be lost (aHR: 2.04; 95% CI: 1.04–4.12), consistent with national-level DR-TB data from South Africa which identifies positive smear microscopy at treatment initiation as a strong predictor of mortality by 24 months[28]. Treatment criteria for receiving decentralized care as outlined in the 2011 South African decentralization guidelines state that[10]: patients should be ambulatory and have a low grade transmission risk (acid fast bacilli (AFB) smear negative). However, these guidelines are not routinely enforced by decentralized clinics as evident in this cohort of enrolled patients of which 25% were smear positive at treatment start. Stricter adherence to national guidelines may then allow for patients with high grade transmission risk (AFB smear positive) to be treated at inpatient facilities with closer monitoring until stable.

Although not reaching statistical significant in the crude or adjusted analysis, HIV infection in combination with ART, was also a risk factor for attrition. We did find that close to half of patients on ART for longer than six months at the time of RR/MDR TB treatment initiation had a viral load ≥1000 copies/ml consistent with virologic failure. The poorer immunological condition of these patients may then increase their risk of poor treatment outcomes.

The highest risk of death in our cohort was during the first three months of RR/MDR-TB treatment, suggesting that there may be a role for closer monitoring of high-risk patients: those with advanced HIV, moderate/severe anaemia, or smear-positive disease, those whose diagnosis of RR/MDR-TB was made in a hospital setting or did not start RR/MDR-TB treatment within a week of diagnosis. Previous studies have demonstrated that decentralized and outpatient care for DR-TB has been crucial to improving access to treatment, reducing the time to treatment initiation, and reducing primary LTFU[5, 29]. It remains unclear, however, how this model of care affects treatment outcomes in those with more advanced disease at presentation who may require the closer monitoring provided in a hospital or sub-acute care setting during the early treatment period. Short term treatment in sub-acute care facilities for patients too sick for outpatient DR-TB treatment has been piloted in the Medecins Sans Frontieres (MSF) program in Khayelitsha, Cape Town with some success, and improving access to this model of care across all decentralized RR/MDR-TB treatment sites may reduce early death and LTFU in the sickest patients[30].

While 12 months is an interim time point in the treatment course in use during this study, it is the endpoint of the 9–12 month short course recommendation made by the WHO in 2016[31], providing early evidence of what to expect with the new regimen. The 9–12 month short course RR/MDR-TB regimen endorsed by the WHO in 2016 is predicted to improve outcomes in part by reducing LTFU when compared to traditional long course treatment duration[31]. Although death and LTFU was most prominent in the first three months after treatment initiation, both outcomes continued throughout the treatment period. While short-course regimen achieved 78.1% treatment success under clinical trial conditions[32], the impact of the shortened regimen on treatment outcomes in routine care is not yet known. Based on our study findings of 31% death and LTFU by 12 months we predict that rates of attrition will continue to remain unacceptably high despite the shortened regimen in programmatic conditions.

### Limitations

This study was conducted at a single, urban treatment site in Johannesburg, South Africa with a high-rate of HIV co-infection. Findings reported here may not be representative of the entire RR/MDR-TB population in South Africa. We were not able to obtain outcomes for the substantial minority of patients who transferred to other facilities prior to completing 12 months’ follow-up, but outcomes for this group may differ from those in our analysis.

## Conclusion

A third of patients with RR/MDR-TB (31%) in Johannesburg, South Africa died or were LTFU by 12 months after RR/MDR-TB treatment initiation. Early attrition was related to baseline disease severity, namely moderate and severe anaemia and smear positive pulmonary disease. High rates of attrition by 12 months, the halfway point of RR/MDR-TB treatment, suggest that earlier monitoring, before the 24-month regimen endpoint, can alert clinicians to attrition risks while patients remain in care. High rates of death and LTFU at 12 months also suggest that attrition will remain unacceptably high during the newer short-course DR-TB treatment regimen endorsed by the WHO in 2016 unless it is accompanied by interventions targeting early death and LTFU.
